# Early reduction of SARS-CoV-2-replication in bronchial epithelium by kinin B_2_ receptor antagonism

**DOI:** 10.1007/s00109-022-02182-7

**Published:** 2022-03-05

**Authors:** Constanze A. Jakwerth, Martin Feuerherd, Ferdinand M. Guerth, Madlen Oelsner, Linda Schellhammer, Johanna Giglberger, Lisa Pechtold, Claudia Jerin, Luisa Kugler, Carolin Mogler, Bernhard Haller, Anna Erb, Barbara Wollenberg, Christoph D. Spinner, Thorsten Buch, Ulrike Protzer, Carsten B. Schmidt-Weber, Ulrich M. Zissler, Adam M. Chaker

**Affiliations:** 1 Center of Allergy & Environment (ZAUM), Technical University of Munich and Helmholtz Center Munich, German, Research Center for Environmental Health, Member of the German Center for Lung Research (DZL), CPC-M, and Member of the Helmholtz I&I Initiative, Biedersteiner Str. 29, 80202 Munich, Germany; 2 Institute of Virology, Technical University of Munich/Helmholtz Zentrum München, German Center of Infectiology Research (DZIF), Munich partner site, Munich, Germany; 3 Institute of Laboratory Animal Science, University of Zurich, Zurich, Switzerland; 4 Institute of Pathology, Technical University Munich, Munich, Germany; 5 Institute of Medical Informatics, Statistics and Epidemiology, Medical School, Technical University of Munich, Munich, Germany; 6 Department of Otorhinolaryngology and Head and Neck Surgery, Medical School, Technical University of Munich, Munich, Germany; 7 Department of Internal Medicine II, University Hospital Rechts Der Isar, Medical School, Technical University of Munich, Munich, Germany

**Keywords:** ACE2, COVID-19, Kinin, B_2_R-antagonist, Kinin-kallikrein-system, SARS-CoV-2

## Abstract

**Abstract:**

SARS-CoV-2 has evolved to enter the host via the ACE2 receptor which is part of the kinin-kallikrein pathway. This complex pathway is only poorly understood in context of immune regulation but critical to control infection. This study examines SARS-CoV-2-infection and epithelial mechanisms of the kinin-kallikrein-system at the kinin B_2_ receptor level in SARS-CoV-2-infection that is of direct translational relevance. From acute SARS-CoV-2-positive study participants and -negative controls, transcriptomes of nasal curettages were analyzed. Primary airway epithelial cells (NHBEs) were infected with SARS-CoV-2 and treated with the approved B_2_R-antagonist icatibant. SARS-CoV-2 RNA RT-qPCR, cytotoxicity assays, plaque assays, and transcriptome analyses were performed. The treatment effect was further studied in a murine airway inflammation model in vivo. Here, we report a broad and strong upregulation of kallikreins and the kinin B_2_ receptor (B_2_R) in the nasal mucosa of acutely symptomatic SARS-CoV-2-positive study participants. A B_2_R-antagonist impeded SARS-CoV-2 replication and spread in NHBEs, as determined in plaque assays on Vero-E6 cells. B_2_R-antagonism reduced the expression of SARS-CoV-2 entry receptor ACE2, G protein–coupled receptor signaling, and ion transport in vitro and in a murine airway inflammation in vivo model. In summary, this study provides evidence that treatment with B_2_R-antagonists protects airway epithelial cells from SARS-CoV-2 by inhibiting its replication and spread, through the reduction of ACE2 levels and the interference with several cellular signaling processes. Future clinical studies need to shed light on the airway protection potential of approved B_2_R-antagonists, like icatibant, in the treatment of early-stage COVID-19.

**Graphical Abstract:**

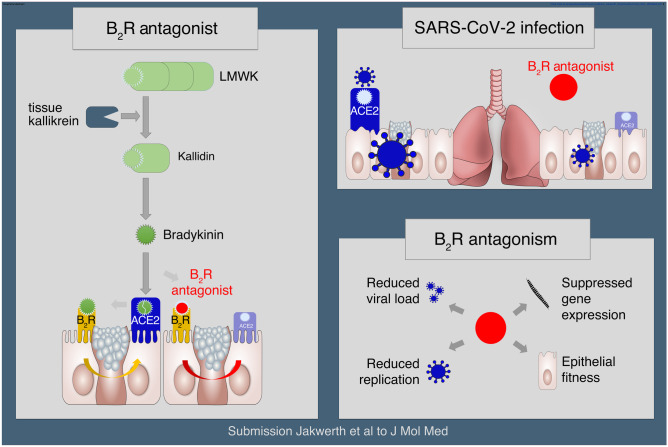

**Key messages:**

- Induction of kinin B_2_ receptor in the nose of SARS-CoV-2-positive patients.
- Treatment with B_2_R-antagonist protects airway epithelial cells from SARS-CoV-2.
- B_2_R-antagonist reduces ACE2 levels in vivo and ex vivo.
- Protection by B_2_R-antagonist is mediated by inhibiting viral replication and spread.

**Supplementary information:**

The online version contains supplementary material available at 10.1007/s00109-022-02182-7.

## Introduction

SARS-CoV-2 vaccines have been approved worldwide since the end of 2020 and are starting to show their protective effects in public health [1, 2]. Even with vaccines at hand, an important medical need for therapeutic approaches for COVID-19 remains: immunocompromised individuals may not mount a sufficient immune response after vaccination and escape variants, such as the currently spreading SARS-CoV-2 variant Omicron [3], may breach protection afforded by the vaccines [4–7].

Key factors for SARS-CoV-2 cell entry are two cell surface molecules, angiotensin-converting enzyme 2 (ACE2) and transmembrane serine protease (TMPRSS)2 [8]. TMPRSS2 cleaves the coronaviral spike protein and primes it for cell fusion, while ACE2 enables the virus particle to enter the cell by binding of its spike protein [9, 10]. The latter acts as central component in its function as terminal carboxypeptidase in the counter-regulatory axis of the renin-angiotensin-system (RAS) and the contact-activation-system (CAS) [8, 11], which initiates blood coagulation and can additionally activate the kinin-kallikrein-system (KKS) [12]. In its role in the RAS, ACE2 has anti-vasoconstrictive and anti-inflammatory effects by hydrolyzing the vasoconstrictive and tissue-damaging angiotensin II, which contributes to airway remodeling and fibrosis [13, 14], to angiotensin (1–7) [15]. In its role in the KKS, ACE2 further hydrolyzes vasoactive peptides such as des-Arg9-bradykinin (DABK), which activates the pro-inflammatory axis of the KKS [16] via the inducible kinin B_1_ receptor (*BDKRB1*;B_1_ receptor;B_1_R) [17]. While DABK is the ligand of B_1_R, bradykinin, the end product of the KKS-cascade, activates the constitutively expressed kinin B_2_ receptor (*BDKRB2*;B_2_ receptor;B_2_R) [18]. Through this mechanism, bradykinin mediates its pro-inflammatory effects by eliciting a variety of responses, including vasodilation and edema, via the G protein–triggered phosphatidylinositol-calcium second messenger-system [19–23]. The fact that SARS-CoV-2 utilizes ACE2 to enter airway cells along with the fact that ACE2 is a multifunctional enzyme that counter-regulates the ACE-driven mechanisms of the RAS and balances the KKS may therefore explain the serious course of COVID-19, not only in the lungs but systemically [24, 25].

Recent publications suggest that the KKS could play a role in COVID-19. KKS comes into play particularly in connection with the high prevalence of thromboembolic events in severely ill COVID-19 patients [7, 17, 26–28]. A recent study on a cohort of 66 COVID-19 patients admitted to the intensive care unit showed that the KKS was strongly activated, which was reflected in the consumption of factor XII (*F12*), pre-kallikrein (*KLKB1*), and high-molecular-weight-kininogen (HMWK; *KNG1*) [26]. When activated, plasma-kallikrein (*KLKB1*) releases kinins from HMWK (*KNG1*) in the peripheral blood. In tissues, however, the functional *real* tissue kallikrein (*KLK1*) generates bradykinin and kallidin [29], but by cleavage of low-molecular-weight-kininogen [30], which is an additional splice product of the *KNG1* gene [31, 32]. It has further been hypothesized that kinin-dependent “local lung angioedema” involving B_1_R and B_2_R is an important characteristic of COVID-19 [33–36]. This study examines the potential of an intervention in the KKS at the kinin receptor level in SARS-CoV-2-infection with translational relevance and reveals an antiviral and protective effect of B_2_R-antagonism on human bronchial epithelium.

## Materials and methods

### Human study participants and nasal brushings

Nasal brushings were performed as part of a larger healthcare professional observational cohort study, which was approved by the Ethics Commission of the Technical University of Munich (AZ 175/20 s) during the first COVID-19 wave in Germany in 2020. Nasal brushings were obtained from 7 healthy healthcare professionals and 4 healthcare professionals with new onset of mild to moderate respiratory symptoms and within 2 days of newly confirmed SARS-CoV-2 diagnosis. No vaccine or specific treatment was available at the time of sampling. RNA was extracted from these nasal brushings and subjected to whole-genome transcriptome analysis (see Supp.Info.). All participants gave written informed consent prior to participation (Table 1).

**Table 1:** Demographic data of healthcare professional cohort

**Parameter**	**Negative** (***n*** = **7**)	**Positive acute** (***n*** **=** **4**)	***p***-**value**
Age (years)*	35.86 ± 3.86	37.50 ± 8.78	n.s
Sex (m/f)	2/5	1/3	
IgM (ng/mL)	1.32 ± 0.28	4.34 ± 2.81	n.s
IgG (ng/mL)	0.48 ± 0.08	67.08 ± 19.51	0.0061

*Negative*, tested negative in SARS-CoV-2 qPCR; *positive acute*, tested positive in SARS-CoV-2 qPCR; *IgM*, immunoglobulin M; *IgG*, immunoglobulin G; *n.s.*, not significant; values are depicted as mean ± s.e.m

^*^At informed consent procedure and inclusion into study

### In vivo mouse study

Mice received murine IL-12Fc (1 μg protein in 50μL PBS) or PBS control intranasally [37]. Intranasal application was performed under isoflurane anesthesia in two steps of 25μL per nostril. Forty-eight hours later, the mice received a single subcutaneous injection of icatibant (2 nmol per 10 g body weight; HOE-140 ((icatibant), H157, SLBX4410, Sigma) or PBS control. The experiment was terminated by CO_2_ asphyxiation 6 h or 24 h after injection of icatibant. The experiment was carried out twice. Organs were snap frozen for protein extraction. Experiments were pre-registered at www.animalstudyregistry.org (study title “Effect of drug on ACE2 levels in mice”; 10.17590/asr.0000225). Mice enrolled in the experiment were 6–8 weeks old, from either C57BL/6 J, BALB/c, or C3H HeN strains. Both sexes were included for each strain and means of each mouse type (strain/sex) are depicted as single values in Fig. 2A: circle:female; triangle:male. Black:C57BL/6, midgrey:C3H HeN, light gray:BALB/c strain. Experiments were performed and analyzed in a randomized and blinded fashion. Animals were obtained from Janvier Labs (Le Genest-Saint-Isle, France) and housed 5 per cage and sex in individually ventilated cages at Laboratory Animal Service Center of the University of Zurich in Schlieren (Schlieren, Zurich, Switzerland). The animal vivarium was a specific-pathogen-free (SPF) holding room, that was temperature- and humidity-controlled (21 ± 3 °C, 50 ± 10%), with a 12-h light/dark cycle. All animals had ad libitum access to the same food and water throughout the entire study. All procedures described in this study had previously been approved by the Cantonal Veterinarian’s Office of Zurich, Switzerland (License ZH096/20), and every effort was made to minimize the number of animals used and their suffering.

Additional methods are provided in the supporting information.

## Results

### B_2_ receptor antagonist inhibits replication and spread of SARS-CoV-2

ACE2 is the central viral entry receptor for SARS-CoV-2 on human epithelial cells of the respiratory tract [8]. Recent studies showed that this receptor and its co-receptors are not only expressed in the lower airways, and thus on alveolar epithelial cells type-I and -II, but are also present in the upper airways, but predominantly in the nasal mucosa [38].

**Fig. 1:**
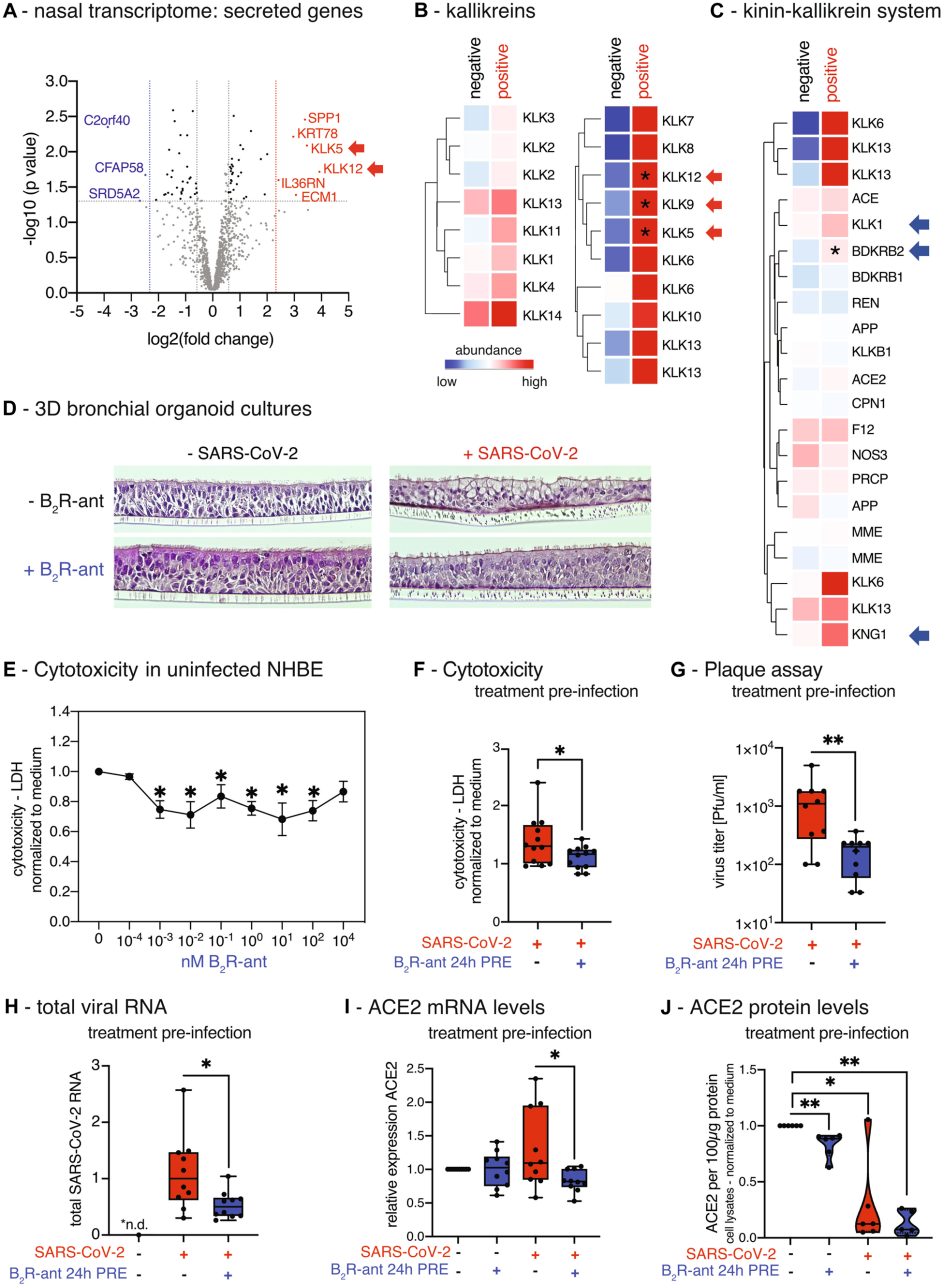
Induction of kallikreins and kinin receptor B_2_ in the nasal mucosa of acutely positive COVID-19 study participants. **A** Volcano plot of significantly differentially regulated genes (DEGs = differentially expressed genes) in nasal curettages of study participants that were acute positive for SARS-CoV-2 compared to healthy individuals (negative) using human miR microarray technology. Highlighted genes have a fold change (FC) ≥ 10 with *P* < 0.05; genes in red are upregulated; genes in blue are downregulated. **B** Heat map of gene expression analysis of kallikrein genes and **C** of genes of the kinin-kallikrein-system (KKS) in nasal curettages comparing acute SARS-CoV-2-positive study participants to healthy controls. All entities are shown. Asterisks indicate significantly regulated genes (*P* < 0.05) in SARS-CoV-2-infected NHBEs compared to medium. Color code indicates Log2-fold change from low (blue) through 0 (white) to high (red). Duplicate gene names indicate the abundance of two or more isoforms of the same gene in the analysis. **D** 3D-air–liquid interphase cultures from NHBEs were pre-treated for 24 h with/without 1 nM B_2_R-antagonist from the basal side and subsequently infected with SARS-CoV-2 for 48 h from the apical side. **E** Lactate dehydrogenase (LDH) cytotoxicity assay using the LDH Cytotoxicity Detection Kit PLUS studying the effect of increasing doses of the B_2_R-antagonist after 48 h in primary NHBEs from 4 donors. Results are depicted as mean ± s.e.m. Statistical tests compared each dose of B_2_R-antagonist with 0 nM B_2_R-antagonist. **F** Cytotoxicity assay determining LDH release into the supernatants of cultures of SARS-CoV-2-infected NHBEs from 12 donors that were pre-treated for 24 h with/without 1 nM B_2_R-antagonist. **G** Quantification of infectious particles in the supernatants of SARS-CoV-2-infected NHBEs from 10 donors that were pre-treated with/without 1 nM B_2_R-antagonist for 24 h. Supernatants were titrated on Vero-E6 cells and plaque assay was quantified 24 h later. Results are depicted as plaque-forming units (PFU) per milliliter. **H** qPCR analysis of total SARS-CoV-2 RNA (viral genome and transcripts, which all contain the N1 sequence region) normalized to human *ACTB* of SARS-CoV-2-infected primary NHBE after 24 h of pre-treatment with/without 1 nM B_2_R-antagonist followed by 24 h of SARS-CoV-2 inoculation. For Fig. 1E, F, and H, statistical tests compared SARS-CoV-2-infected versus uninfected samples or B_2_R-antagonist-treated versus untreated samples. **I** Analysis of human ACE2 gene expression using qPCR (*n* = 10) and **J** of human ACE2 protein levels analyzed by ELISA from cell lysates (*n* = 6) after 24 h of pre-treatment of NHBEs with/without 1 nM B_2_R-antagonist, followed by SARS-CoV-2 inoculation for 24 h

To investigate local effects of the acute SARS-CoV-2-infection on the nasal epithelium, we analyzed the transcriptome of nasal curettages from symptomatic study participants, who tested acutely positive for SARS-CoV-2 (*n* = 4), and from SARS-CoV-2-negative study participants (*n* = 7). In a transcriptome analysis, the most strongly induced genes encoding secreted factors included many members of the kallikrein family (Fig. 1A, Table S1), in particular the kallikreins *KLK5*, *KLK9*, and *KLK12* (Fig. 1B, Table S2). Next, we focused on the central factors of the tissue-KKS as stated above. Two-thirds of the genes were upregulated including the precursor of bradykinin LMWK (*KNG1*), *true* tissue kallikrein (*KLK1*), responsible of hydrolyzing LMWK to kallidin/bradykinin, and further the receptor for bradykinin, B_2_R (*BDKRB2*), which was significantly increased (Fig. 1C, Table S3). Since the two plasma factors factor XII (*F12*) and pre-kallikrein (*KLKB1*) are mainly processed and act in the plasma-KKS, it was expected that these factors are not differentially expressed in the nasal mucosa. The induction of *KNG1*, *KLK1*, and *BDKRB2* in primary nasal samples of SARS-CoV-2-positive study participants is evidence for an autocrine bradykinin effect via B_2_R that is triggered locally during COVID-19 disease.

This finding prompted us to investigate selective kinin B_2_ receptor antagonism in connection with SARS-CoV-2-infection. We therefore hypothesized that a B_2_R-antagonist like icatibant, an approved compound for the treatment of hereditary angioedema [39], counter-regulates the effects of bradykinin during a SARS-CoV-2-infection and thereby has a protective effect on the integrity of the airway mucosa. To circumvent limitations of cell lines like Vero-E6, A549, or Calu-3 cells that are intrinsically impaired to form an interferon response upon viral infection [40], we infected primary human NHBEs with SARS-CoV-2.

To examine the effects of SARS-CoV-2-infection and B_2_R-antagonist treatment on the microscopic integrity of the airway epithelium, 3D-air–liquid interphase organoid cultures were differentiated from primary NHBEs (Supp.Info). After complete differentiation, epithelia were pre-treated from the basal side with the approved B_2_R-antagonist icatibant, and subsequently infected with SARS-CoV-2 from the apical side. The cultures pre-treated with B_2_R-antagonist showed less virus-induced balloon-like structures compared to untreated cultures. The epithelial layers remained qualitatively more intact, which indicates a protective effect of the B_2_R-antagonist for the bronchial epithelium (Fig. 1D). This finding was further strengthened by cytotoxicity assays: the B_2_R-antagonist had no toxic effects on NHBEs even at high doses determined by lactate dehydrogenase (LDH) release, but rather exhibited a cell-protecting effect in uninfected cells (Fig. 1E) and during SARS-CoV-2-infection (Fig. 1F). Next, the supernatants of pre-treated, infected primary NHBEs were collected and titrated onto fresh Vero-E6 cell cultures and plaque assays were performed. Strikingly, we found that in vitro treatment of NHBEs with B_2_R-antagonist prior to infection reduced the number of plaque-forming units (PFU) in a plaque assay by 87% (Fig. 1G). The levels of total SARS-CoV-2-RNA in cells that had been pre-treated with the B_2_R-antagonist decreased by 52% compared to untreated infected NHBEs (Fig. 1H). With regard to the virus entry process, ACE2 was reduced by pre-treatment with B_2_R-antagonist at the mRNA level (Fig. 1I), but just in trend at the protein level (Fig. 1J). However, ACE2 protein levels were significantly reduced upon SARS-CoV-2-infection. The membrane-standing protease TMPRSS2 cleaves the spike protein for SARS-CoV-2 and primes it for optimized binding to its entry receptor ACE2. In contrast to ACE2, *TMPRSS2* transcript levels were significantly increased in infected compared to uninfected NHBEs but were not affected by B_2_R-antagonist pre-treatment (Fig. S2A). Further experiments on the B_2_R-antagonist effect on the SARS-CoV-2-infection of NHBE showed that pre-treatment with B_2_R-antagonist significantly reduced infection-mediated cytotoxicity measured by LDH release (Fig. 1F).

### Repetitive treatment with B_2_R-antagonist inhibits SARS-CoV-2-replication and spread post-infection

**Fig. 2:**
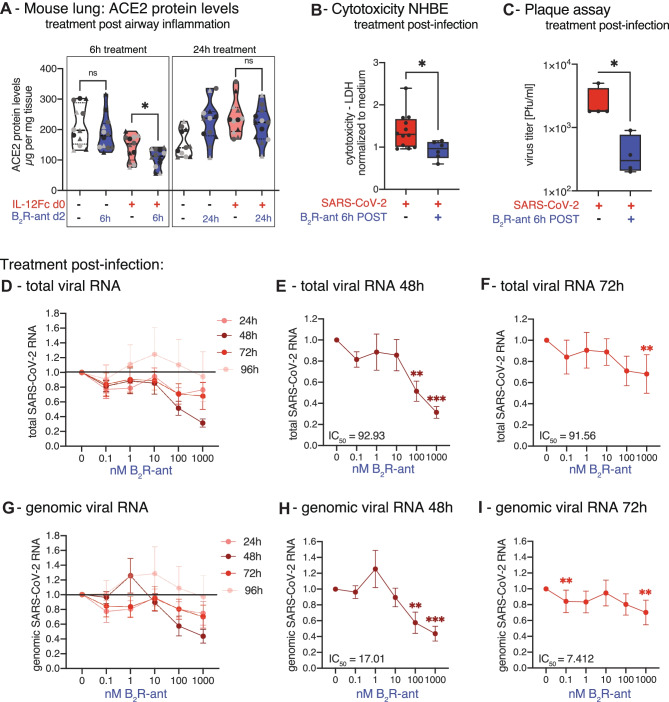
Treatment of NHBE with B_2_R-antagonist post-infection in repeated doses inhibits SARS-CoV-2 replication. **A** In vivo mouse study. Twelve sex-matched mice from three different strains per group were treated on day 0 with intranasal application of 1 μg murine IL-12Fc per mouse or PBS as control to mimic virus-induced airway inflammation. After 48 h, mice were injected s.c. with 2 nmol of the B_2_R-antagonist icatibant per 10 g of body weight or PBS as control. The experiment was terminated either 6 h or 24 h later and murine lung ACE2 protein levels were analyzed by mouse ACE2 ELISA analysis. Circle:female; triangle:male. Black:C57BL/6, mid gray:C3H HeN, light gray:BALB/c strain. The experiment was carried out twice and the data in the figure represent the mean of each mouse type (strain/sex) of both experiments. Statistical tests compared B_2_R-antagonist-treated versus untreated groups. **B** Cytotoxicity assay determining LDH in supernatants from SARS-CoV-2-infected NHBEs from 12 donors that were treated with/without 1 nM B_2_R-antagonist after 6 h of infection for another 24 h. **C** Quantification of infectious particles in the supernatants from SARS-CoV-2-infected NHBEs from 4 donors that were treated with/without 1 nM B_2_R-antagonist after 6 h of infection for another 24 h. Supernatants were titrated on Vero-E6 cells and plaque assay was quantified 24 h later. Results are depicted as plaque-forming units (PFU) per milliliter. For Fig. 2B–C, statistical tests compared B_2_R-antagonist-treated versus untreated samples. **D** Relative quantification of total SARS-CoV-2 RNA (viral genome and transcripts, which all contain the N1 sequence region) and **G** genomic SARS-CoV-2 RNA (containing the *RdRP* gene) normalized to housekeeping gene index of human *ACTB*, *HPRT*, *18S* in NHBEs from 8 independent donors that were infected with SARS-CoV-2 for 6 h and then treated with increasing doses of the B_2_R-antagonist icatibant repeatedly every 24 h for a total of 96 h. In cells treated with **E** 100 nM and **F** 1000 nM icatibant for 48 h and with **H** 100 nM and **I** 1000 nM icatibant for 72 h, total SARS-CoV-2 RNA and genomic SARS-CoV-2 RNA were significantly reduced. Red indicates SARS-CoV-2-infection; blue indicates B_2_R-antagonist treatment. PRE indicates pre-treatment; POST indicates post-treatment. In Fig. 2D–I, results are depicted as mean ± s.e.m. and statistical tests compared each dose of icatibant with 0 nM icatibant. Statistically significant differences were depicted as *p*-values *P < 0.05, **P < 0.01, and ***P < 0.001. ns indicates non-significant. + infected/treated;—indicates not infected/not treated

The finding that B_2_R-antagonism leads to a downregulation of ACE2 protein levels in lung epithelial cells was confirmed in vivo in a murine airway inflammation model. To mirror COVID-19 pathogenesis, mice were treated with IL-12, which mimics virus-induced airway inflammation via activation of the IL-12/IFN-γ-axis [37, 41]. Specifically, mice received intranasal murine IL-12Fc, to generate a pro-inflammatory state in the lungs. The experiment was designed in two blocks of 24 sex-matched mice from three different strains per group, to rule out any confounding genetic effect. After 48 h, mice were injected subcutaneously (s.c.) with the B_2_R-antagonist and the experiment was terminated 6 h or 24 h later to analyze ACE2 protein levels in the lungs. IL-12Fc pre-treated mice, which were then further treated with the B_2_R-antagonist on day 2, showed reduced ACE2 protein levels in the lungs after 6 h compared to control mice, which were only treated with PBS on day 2 (Fig. 2A). This effect decreased after 24 h.

Anticipating treatment of SARS-CoV-2-infected study participants with the B_2_R-antagonist icatibant, NHBEs were first infected with SARS-CoV-2 and then treated with the B_2_R-antagonist 6 h after infection. Confirming the results of pre-treatment, post-infection treatment with the B_2_R-antagonist also attenuated the cytopathic effect of SARS-CoV-2 (Fig. 2B) and reduced the number of PFU in a plaque assay on Vero-E6 cells by 84% (Fig. 2C).

We also aimed to reflect repeated dosage [42] during treatment of early infection by treating NHBEs post-infection every 24 h with B_2_R-antagonist repeatedly for a period of 96 h, reflecting the drug administration of this particular substance in real life. In cells treated post-infection with 100 nM icatibant for 48 h, total viral RNA (Fig. 2D–F IC_50_(total RNA 48 h) = 92.93; IC_50_(total RNA 72 h) = 91.56) and also genomic viral RNA (Fig. 2G–I; IC_50_(total RNA 48 h) = 17.01; IC_50_(total RNA 72 h) = 7.412) were significantly reduced by 49% and 42% on average, respectively. Treatment with 1000 nM icatibant for 48 h led to a reduction of total SARS-CoV-2 RNA (Fig. 2D–F) and also of genomic SARS-CoV-2 RNA (Fig. 2G–I) by 69% and 56% on average, respectively. Genomic viral RNA was detected using RT-qPCR against the sequence of the SARS-CoV-2 RNA-dependent RNA polymerase (*RdRP*), which is only found in virions and during the viral replication. On the other hand, total viral RNA was detected with qPCR targeting a sequence of the SARS-CoV-2 N gene that is present in the viral genome and also in every SARS-CoV-2 protein-encoding transcript. Both, total SARS-CoV-2 RNA and genomic viral RNA levels were reduced upon treatment with the B_2_R-antagonist (Fig. 2D–I).

### B_2_ receptor antagonism broadly silences gene expression in bronchial epithelial cells while maintaining cell-intrinsic antiviral response

**Fig. 3:**
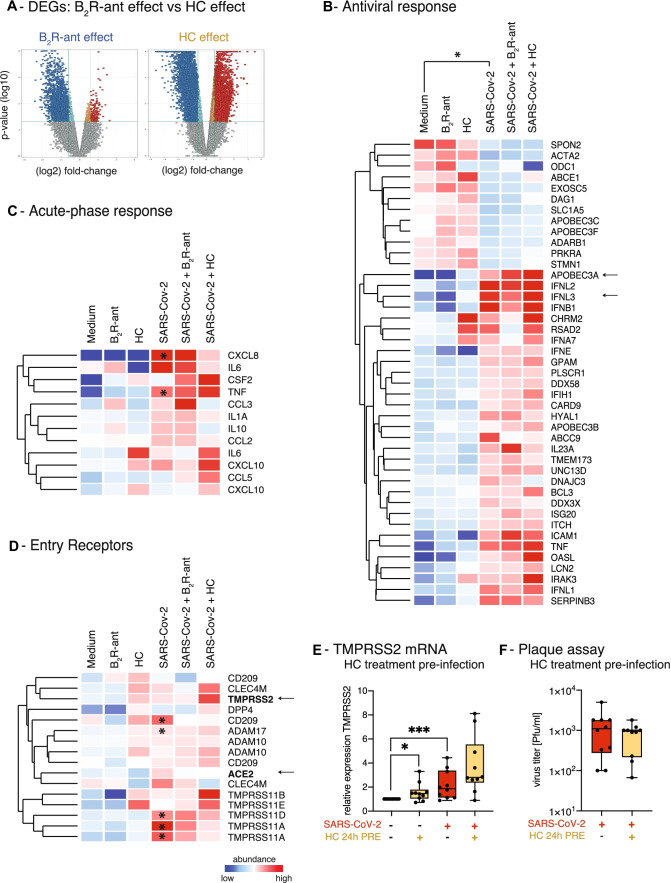
B_2_R-antagonism exhibits a protective and suppressive effect on gene expression profile of airway epithelial cells. **A** Volcano plots showing global gene expression changes induced by either treatment with B_2_R-antagonist or hydrocortisone (HC). Red indicates significantly upregulated entities; blue indicates significantly downregulated entities. Gene expression analysis of pre-treated NHBEs after 24 h of SARS-CoV-2-infection. **B** Heat map of gene expression analysis of genes involved in the epithelial antiviral response, analysis of the effects of SARS-CoV-2-infection. Only entities with significant changes between SARS-CoV-2-infection and medium are shown (gene expression fold change FC ≥ 1.5 with *P* < 0.05). **C** Heat map of gene expression analysis of genes involved in the acute-phase response is depicted. All entities are shown. Asterisks indicate significantly regulated genes (*P* < 0.05) in SARS-CoV-2 compared to medium. **D** Heat map of gene expression analysis of known and potential virus entry receptors is depicted. All entities are shown. Color code indicates Log2-fold change from low (blue) through 0 (white) to high (red). Asterisks indicate significantly regulated genes (*P* < 0.05) in SARS-CoV-2-infected NHBEs compared to medium. Duplicate gene names indicate the presence of two or more isoforms of the same gene in the analysis. **E** Analysis of *TMPRSS2* gene expression by qPCR after 24 h of pre-treatment with/without 10 μM hydrocortisone (HC) followed by 24 h of SARS-CoV-2 inoculation. Red indicates SARS-CoV-2-infection; yellow indicates pre-treatment with hydrocortisone (HC). Statistical tests compared SARS-CoV-2-infected versus uninfected samples or B_2_R-antagonist-treated versus untreated samples. **F** Quantification of infectious particles in the supernatants of SARS-CoV-2-infected NHBEs from 10 donors that were pre-treated with/without 10 μM hydrocortisone (HC) for 24 h. Supernatants were titrated on Vero-E6 cells. The plaque assay was quantified 24 h later. Results are depicted as plaque-forming units (PFU) per milliliter

Severe cases of COVID-19 develop cytokine storms [43–45] characterized by excessive systemic release of multiple cytokines including IP-10 (*CXCL10*), IL-6, IL-8 (*CXCL8*), and IL-10 [46–49]. These cases are currently treated with immunomodulating drugs, such as corticosteroids or biologics, like tocilizumab [50], though these treatments may interfere with or alter the antiviral immune response. We therefore compared the effect of B_2_R-antagonism on gene expression of SARS-CoV-2-infected bronchial epithelium with the effect of hydrocortisone. While the B_2_R-antagonist mainly suppressed epithelial gene expression during infection, the effects of hydrocortisone on gene induction and gene repression were comparable (Fig. 3A, Tables S4-5). This finding matches previous reports [51].

With regard to cell-intrinsic antiviral immunity, differentially expressed genes (DEGs) in NHBEs induced by SARS-CoV-2-infection included type-I and -III interferons and IFN-inducible, antiviral *APOBEC* genes (Fig. S1E, Table S6). SARS-CoV-2-infection particularly induced antiviral cytidine deaminases APOBEC3A and B, which we previously described to be induced by type-I interferons in the treatment of hepatitis B-virus-infection [52]. *APOBEC3C* mRNA levels, however, were decreased in SARS-CoV-2-infected NHBE, which could indicate a novel evasion mechanism [53]. Neither B_2_R-antagonist nor hydrocortisone inhibited the expression of genes with cell-intrinsic antiviral effects, but even increased the antiviral factor *APOBEC3A* at the mRNA level (Fig. 3B, Table S7) [54].

Our gene expression analysis shows that SARS-CoV-2-infection further induces the expression of acute-phase proteins, such as TNF-α and IL-8 (*CXCL8*) [55, 56], as well as IL-17C, MIP-3α (*CCL20*), IL-36γ [57], and chemokines CXCL1,-2,-3,-8,-17, CCL2,-3,-5 [57] in primary airway epithelial cells (Fig. 3C, Table S8). The induction of these factors most likely contributes to the recruitment and activation of relevant immune cells to the site of infection. In addition, gene expression of acute-phase proteins was not significantly affected in airway epithelial cells by B_2_R-antagonist or hydrocortisone treatment (Fig. 3C, Table S8). Neither drug interfered with cell-intrinsic antiviral immune mechanisms, like IFN induction, APOBEC induction, or chemokine induction, thereby showing great potential for treatment options of COVID-19 while maintaining the host’s antiviral immune response.

In addition, we found that SARS-CoV-2-infection increases the expression of three known and postulated entry (co-)receptors: (1) transmembrane serine protease TMPRSS11A (Fig. S1C, Table S6, 9–13), which was described to prime the MERS coronavirus spike protein [58], (2) transmembrane serine protease TMPRSS11D, which was shown to activate SARS-CoV-2 spike protein [59], and (3) pathogen-associated molecular pattern-binding C-type lectin receptor DC-SIGN (*CD209*), which was described to serve as entry receptor for SARS-CoV [60] and has also been suggested as a receptor for SARS-CoV-2. The induction of these additional entry receptor candidates triggered by SARS-CoV-2-infection may potentiate the viral spread in the bronchial epithelium and thus represent a pathogenetic mechanism that needs further research.

Overall, treatment with the B_2_R-antagonist and hydrocortisone had no significant effects on the expression of most candidate viral entry receptors, except for hydrocortisone, which enhanced the expression of TMPRSS proteases (Fig. 3D, Table S14). In particular, when focusing on the known SARS-CoV-2 entry receptors, hydrocortisone treatment of uninfected cells was already sufficient to induce an increase in *TMPRSS2* gene expression (Fig. 3E). SARS-CoV-2-infection per se also increased *TMPRSS2* expression, and pre-treatment of SARS-CoV-2-infected NHBEs with hydrocortisone further potentiated this effect. On the other hand, *ACE2* expression showed only a slight upward trend after hydrocortisone pre-treatment (Fig. S2B). Finally, hydrocortisone pre-treatment of SARS-CoV-2-infected NHBEs had no inhibitory effect on the release of infectious particles 24 h after infection (Fig. 3F), which was expected, since treatment of COVID-19 study participants with corticosteroids has an immunomodulatory rationale.

### B_2_ receptor antagonist counter-balances virus-induced gene expression, particularly genes involved in G protein–coupled receptor (GPCR) signaling and ion transport

**Fig. 4:**
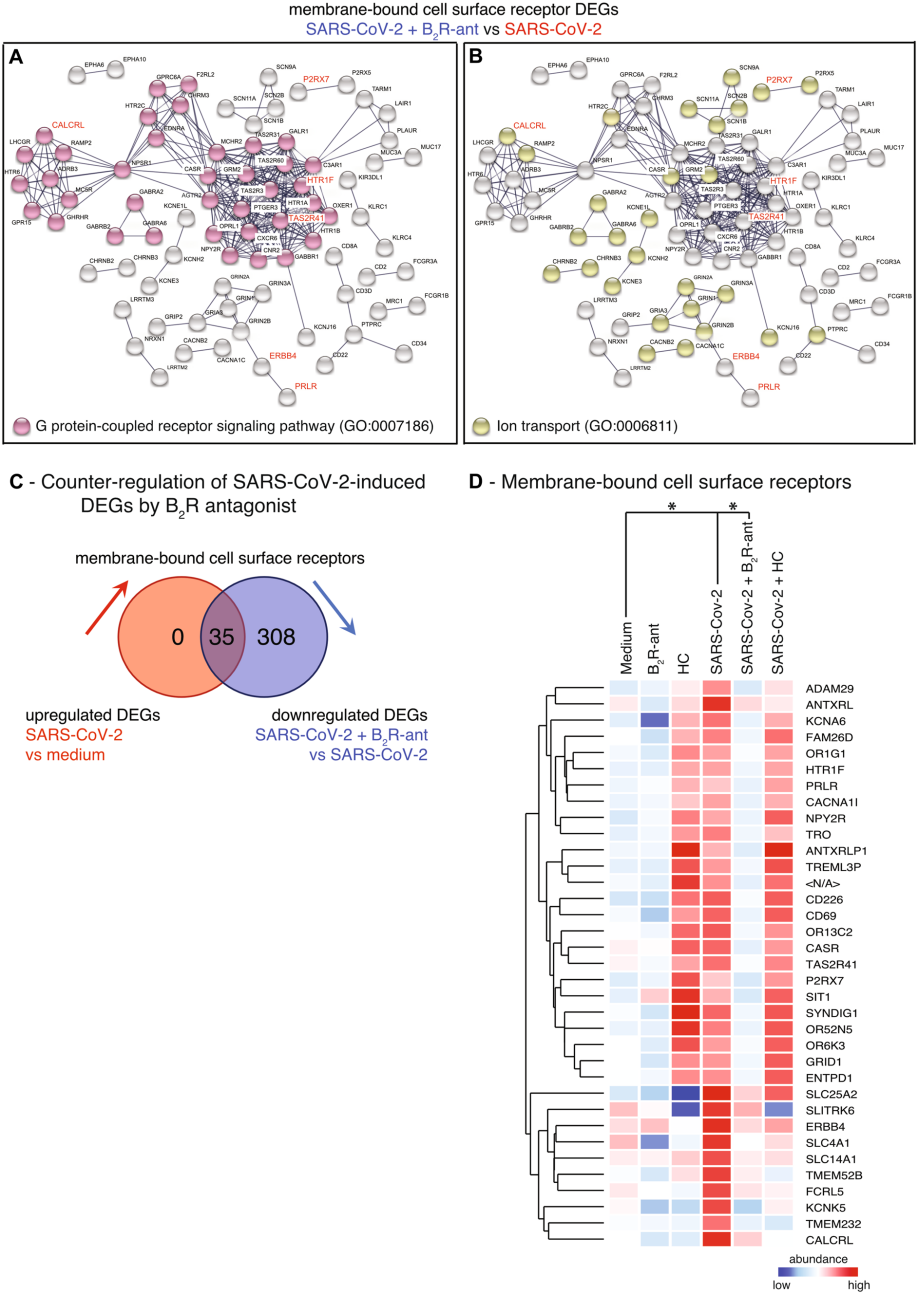
B_2_R-antagonism exhibits a protective and suppressive effect on gene expression profile of airway epithelial cells. GO-term enrichment analysis, which results from the string network analysis of significant DEGs from the gene expression analysis comparing infected NHBE pre-treated with B_2_R-antagonist with untreated infected NHBE (SARS-CoV-2 + B_2_R-antagonist versus SARS-CoV-2). Depicted are enrichment of **A** GO-term GO:0,007,186 “G protein-coupled receptor signaling pathway” and **B** GO-term GO:0,006,811 “Ion transport.” Genes that were significantly upregulated in the comparison SARS-CoV-2 versus medium are highlighted in red. **C** Venn diagram showing the cut set of upregulated membrane-bound cell surface receptors in SARS-CoV-2 versus medium and of downregulated DEGs in SARS-CoV-2 + icatibant versus SARS-CoV-2 (FC ≥ 1.5; *P* ≤ 0.05). **D** Heat map of gene expression analysis of the 35 membrane-bound cell surface receptors defined in cut set from Fig. 4C, all upregulated upon SARS-CoV-2-infection and downregulated upon pre-treatment with B_2_R-antagonist are depicted. Only entities with significant changes between SARS-CoV-2-infection and medium (up) and between SARS-CoV-2 + B_2_R-antagonist and SARS-CoV-2 (down) are shown (gene expression fold change FC ≥ 1.5 with *P* < 0.05). Color code indicates Log2-fold change from low (blue) through 0 (white) to high (red). Duplicate gene names indicate the abundance of two or more isoforms of the same gene in the analysis

In order to identify gene networks that are attenuated by B_2_R-antagonism, DEGs were processed in a network analysis using the database “String” to identify enriched cellular processes. B_2_R-antagonism reduced the expression levels of 343 membrane-bound receptors significantly in treated versus untreated SARS-CoV-2-infected NHBEs (Table S15). Two particular cellular processes affected by pre-treatment with the B_2_R-antagonist were identified, namely G protein–coupled receptor signaling (GO:0,007,186; Fig. 4A, S2C, Tables S4, S17-18) and ion transport (GO:0,006,811; Fig. 4B, Tables S4, S17–18). DEGs involved in both processes were significantly downregulated in treated versus untreated SARS-CoV-2-infected NHBEs (Tables S16-18). Notably, all 35 cell surface receptors induced by SARS-CoV-2-infection were downregulated in cells that were treated with the B_2_R-antagonist (Fig. 4C, D, Tables S19-20).

## Discussion

Here, we provide evidence for the effect of interference with the KKS at the kinin B_2_ receptor level as a means of protecting the airway epithelium from SARS-CoV-2-infection, while maintaining cell-intrinsic antiviral host response.

We initially hypothesized that through KKS interference, either feedback mechanisms or modulated signal transduction targets the virus entry receptor ACE2 and thus interferes with the spread of SARS-CoV-2. To this end, the approved B_2_R-antagonist icatibant was used in this study. We demonstrate that treatment with a B_2_R-antagonist inhibits the replication and spread of SARS-CoV-2 in primary airway epithelial cells, which was determined by a decrease in total and genomic SARS-CoV-2-RNA, resulting in less infectious particles in plaque assays, both when applied pre- and post-infection. While a low concentration of 1 nM B_2_R-antagonist was sufficient to reduce viral RNA in primary bronchial epithelial cells when cells were treated pre-infection, 100 nM B_2_R-antagonist was required to this effect, when cells were treated post-infection. In addition, the significant reduction in virus load as determined by PCR tapered off after 96 h. On the one hand, in vitro infections are performed with excess amounts of virus particles. On the other hand, the fact that, due to its constitution as a peptide analog, the B_2_R-antagonist icatibant used in this study has a short half-life in the human body [42] but also pharmacological tolerance to interference at receptor level may explain why the effect reached significance after 6 h but did not persist. Therefore, it may be required to administer higher doses of the B_2_R-antagonist to COVID-19 patients a few times per day to inhibit viral replication in the long term. Due to this necessary repetitive administration of the B_2_R-antagonist, the monoclonal anti-plasma-kallikrein (*KLKB1*) antibody lanadelumab [61] may also be considered a potential pharmacologic alternative. However, it is not clear whether the effects of the B_2_R blockade alone and its effects on the KKS are responsible for the SARS-CoV-2 inhibition, or whether the compound itself additionally mediates a direct antiviral effect. Therapeutic application of the B_2_R-antagonist icatibant in future dose-finding studies should therefore focus on early intervention with at least two doses daily [62] and on either optimized pharmacokinetics or increased high local tissue concentrations, e.g., through topical application.

Two potential mechanisms of action for suppressing SARS-CoV-2-replication and spread in airway epithelium are revealed by this study:

1. Treatment with the B_2_R-antagonist led to a downregulation of the viral entry receptor ACE2, in vitro in primary airway epithelial cells and in vivo in a murine airway inflammation model. Since the decrease of genomic SARS-CoV-2 RNA and total SARS-CoV-2 RNA was comparable, we conclude that the B_2_R-antagonist icatibant does probably not affect the viral transcription machinery but inhibits the infection rather on the levels of entry, protein synthesis/processing, and assembly, maturation, or budding.
  In comparison, the corticosteroid hydrocortisone even upregulated TMPRSS2 in infected airway epithelial cells. It is noteworthy that hydrocortisone did not change the release of infectious particles from airway epithelial cells into the supernatant. Although TMPRSS2 expression was even enhanced by hydrocortisone, our data implicate that this effect on TMPRSS2 alone is insufficient to increase susceptibility for SARS-CoV-2-infection.
2. Treatment with the B_2_R-antagonist had a broad suppressive effect on gene expression of multiple cell signaling molecules, in particular on membrane-standing factors involved in GPCR signaling and ion transport.
  It has recently been published that SARS-CoV-2 may use cellular GPCR signaling pathways, thereby modulate epithelial transport mechanisms involved in ion transport and thereby cause a local ion imbalance in the airways [63]. In addition, an extensive recent study described that intracellular SARS-CoV-2 protein interactions include factors involved in intracellular trafficking and transport [64]. In fact, SARS-CoV-2-infection led to a differential regulation of the gene expression of 12 potassium channel (5 upregulated/7 downregulated), 1 sodium channel (down), but in particular of 55 members of the solute carrier family (24 downregulated/31 upregulated) in primary airway epithelial cells. On the other hand, B_2_R-antagonist treatment of SARS-CoV-2-infected NHBE resulted in a downregulation of 20 potassium channels and 6 sodium channels, as well as a downregulation of 29 members of the solute carrier family. We therefore conclude that B_2_R-antagonism not only impedes the viral entry process by reducing ACE2, as we had hypothesized, but also counter-regulates cellular processes that include GPCR signaling and transmembrane ion transport, which SARS-CoV-2 may utilize for efficient cell entry, replication, and viral spread.

In conclusion, the results of this study suggest that B_2_ receptor antagonism protects airway epithelial cells from SARS-CoV-2 spread by reducing ACE2 levels and by interfering with several cellular signaling processes. Further research is needed to elucidate more details about molecular mechanisms involved in the viral life cycle that kinin B_2_ receptor antagonism targets and underlie its effects against SARS-CoV-2-infection. Based on these data, we speculate that the protective effects of B_2_R-antagonism could potentially prevent the early stages of COVID-19 from progressing into severe acute respiratory distress syndrome (ARDS) with structural airway damage and fibrotic changes. We therefore propose that the safe approved B_2_R-antagonist icatibant be tested in clinical trials for two important aspects: (1) Treatment of early COVID-19 disease targeting the replication and spread of the virus. (2) Optimized dosage regimen to reflect pharmacokinetics and possible pharmacological tolerance at the receptor level. Future controlled clinical trials must provide substantial evidence for optimal dosage regimen, application, efficacy, and safety to investigate, whether KKS interference at the kinin B_2_ receptor level can prevent the escalation of COVID-19 to ARDS and long-term lung damage.

## Supplementary Information

Below is the link to the electronic supplementary material.

Supplementary file1 (DOCX 40 KB)

Supplementary file2 (EPS 1232 KB)

Supplementary file3 (EPS 699 KB)

## Data Availability

The data discussed in this publication are deposited in NCBI’s Gene Expression Omnibus and are accessible under the GEO Series accession number GSE176405.

## Abbreviations

ACE2: Angiotensin-converting enzyme 2
ant-B_2_R: Kinin B_2_ receptor antagonist
*BDKRB1*: Kinin B_1_ receptor (gene name)
*BDKRB2*: Kinin B_2_ receptor (gene name)
B_1_R: Kinin B_1_ receptor
B_2_R: Kinin B_2_ receptor
B_2_R-antagonist: Kinin B_2_ receptor antagonist
CAS: Contact-activation-system
COVID-19: Coronavirus disease of 2019
DABK: Des-Arg9-bradykinin
GPCR: G protein–coupled receptor
HC: Hydrocortisone
HMWK: High-molecular-weight-kininogen
IC50: Half maximal inhibitory concentration
KKS: Kinin-kallikrein-system
LDH: Lactate dehydrogenase
LMWK: Low-molecular-weight-kininogen
NHBE: Normal human bronchial epithelial cells
RAS: Renin-angiotensin-system
SARS-CoV-2: Severe acute respiratory syndrome coronavirus-2
TMPRSS: Transmembrane serine protease
